# *Methods and Techniques*—A new article category for *Plant‐Environment Interactions*

**DOI:** 10.1002/pei3.10103

**Published:** 2023-02-25

**Authors:** Wayne Dawson

**Affiliations:** ^1^ Department of Biosciences Durham University Durham UK

The Editorial Board of *Plant‐Environment Interactions* (*P‐EI*) is happy to announce a new article category called *Methods and Techniques*. *P‐EI* is still a young journal, although we have now published two full volumes (in 2021 and 2022). Over these past 2 years, we have recognized that some submitted studies may not be focused primarily on a research question or hypothesis, but instead they may be describing the invention, development, or improvement of methodological approaches or equipment that can be used to better understand how plants interact with the abiotic and biotic environment. In response to this, in 2023, we will be launching *Methods and Techniques* as an article category that the authors can choose on submission of their manuscripts.

The guidelines for preparation of manuscripts to be considered under *Methods and Techniques* will be the same as those for research articles, with the same limit on total number of figures and tables combined (maximum of 10). However, the structure of *Methods and Techniques* does not have to adhere to the traditional Introduction—Methods—Results—Discussion format of research articles. Manuscripts submitted under this new category should still have an abstract and will need to provide evidence that the new methods/equipment work as intended. This evidence can involve comparison with other established approaches, presentation of statistical analyses that quantify precision, accuracy and/or repeatability, and visual aids, depending on the method and its applications. In the interest of transparency and reproducible sound science, we also ask that where new protocols, workflows. or equipment are described, all details are made available at submission that would be needed by a third party to reproduce the method. This may include design plans, programming code, or detailed lab protocols, and these details can be submitted as supplementary materials for review. Finally, as with all other article categories in our journal, *Methods and Techniques* submissions need to involve interactions between plants and their abiotic or biotic environment for us to consider the submission for publication.

As an open‐access journal, *P‐EI* is an ideal venue for publication of studies introducing new or improved methodologies, because there is great potential for those new approaches to reach and be used by a wide range of plant and environmental scientists across the globe. We look forward to receiving and reading your *Methods and Techniques* submissions soon.

